# A Scoping Review on Tyrosine Kinase Inhibitors in Cats: Current Evidence and Future Directions

**DOI:** 10.3390/ani13193059

**Published:** 2023-09-29

**Authors:** Žiga Žagar, Jarno M. Schmidt

**Affiliations:** IVC Evidensia Small Animal Clinic Hofheim, 65719 Hofheim am Taunus, Germany

**Keywords:** feline, toceranib phosphate, masitinib mesylate, KIT, HER2, mast cell tumor, mammary carcinoma, squamous cell carcinoma, animal model, comparative oncology

## Abstract

**Simple Summary:**

Oncology is a rapidly advancing field in small-animal medicine, underscoring the importance of innovative therapeutic approaches. Tyrosine kinase inhibitors (TKIs) are drugs that block various important cellular functions and play an important role in treating cancer in people and dogs. However, their role in feline oncology is less established. This comprehensive review surveys the existing literature on tyrosine kinases (TKs) and the use of TKIs in cats, aiming to identify knowledge gaps, speculate on potential indications and lay the groundwork for future investigations. Diverse feline tissues have been examined for various TK expressions. However, although limited evidence exists on the use of TKIs in specific feline tumors, it trails behind progress in human and canine oncology. We believe additional research could identify new uses for these drugs and improve therapeutic options for cats with cancer in the future.

**Abstract:**

Tyrosine kinase inhibitors (TKIs) have become invaluable in the treatment of human and canine malignancies, but their role in feline oncology is less defined. While toceranib phosphate and masitinib mesylate are licensed for use in dogs, no TKI is yet approved for cats. This review systematically maps the research conducted on the expression of tyrosine kinases in neoplastic and non-neoplastic domestic feline tissues, as well as the in vitro/in vivo use of TKIs in domestic cats. We identify and discuss knowledge gaps and speculate on the further research and potential indications for TKI use in cats. A comprehensive search of three electronic databases and relevant paper reference lists identified 139 studies meeting the inclusion criteria. The most commonly identified tumors were mast cell tumors (MCTs), mammary and squamous cell carcinomas and injection-site sarcomas. Based on the current literature, toceranib phosphate appears to be the most efficacious TKI in cats, especially against MCTs. Exploring the clinical use of TKIs in mammary carcinomas holds promise. Despite the progress, currently, the evidence falls short, underscoring the need for further research to discover new indications in feline oncology and to bridge the knowledge gaps between human and feline medicine.

## 1. Introduction

Since the cellular product of *v-src*, the first known oncogene, was identified as a protein kinase in the 1980s [1,2,3], this group of cellular transducing molecules has been sparking great interest in cancer research. Protein kinases are enzymes that play key signaling roles in multiple cellular functions such as differentiation, growth, survival and apoptosis [4]. They act by attaching a phosphate group from an ATP molecule to specific residues on either themselves (autophosphorylation) or downstream substrate proteins (phosphorylation) [5]. Protein kinases are commonly classified according to the amino acids they phosphorylate. The two main classes are tyrosine kinases (TKs), which phosphorylate proteins on tyrosine residues, and serine-threonine kinases, which phosphorylate proteins on serine and/or threonine residues [6]. In mammalian cells, mitogenic pathways largely use signaling through tyrosine phosphorylation [5], hence, TKs are of particular interest in oncology. They are further divided into receptor and non-receptor TKs. Receptor TKs (RTKs) are transmembrane proteins with a ligand-binding extracellular domain, a transmembrane domain and a cytoplasmic catalytic domain. Non-receptor TKs lack the transmembrane domain and are found freely in the cytosol, the nucleus and the inner surface of cell membranes [6]. In health, RTKs require specific ligand binding (e.g., growth factors) for activation. In the absence of specific ligands, RTKs remain inactive. Non-receptor TKs are kept inactive by intracellular inhibitor proteins and lipids, as well as through intramolecular autoinhibition. They are activated through the dissociation of inhibitors, recruitment to transmembrane receptors and trans-phosphorylation by other kinases [7].

Dysregulations in different TKs have been shown in various human [8], canine [9,10,11,12,13,14,15,16] and some feline [17,18,19,20] malignancies. Dysregulated TKs remain constitutively active without appropriate negative regulation, resulting in uncontrolled cell proliferation and survival [21]. The therapeutic targeting of TKs in cancer patients can be achieved using either monoclonal antibodies (mAbs) or “small molecule tyrosine kinase inhibitors” [21]. While mAbs are commonly used in human oncology, their use in companion animals is yet to be established [22]. Despite over 40 different tyrosine kinase inhibitors (TKIs) being approved for use in humans [23], only two TKIs, masitinib mesylate (masitinib) [24] and toceranib phosphate (toceranib) [25], have been developed and approved as cancer drugs for dogs. To date, no such drug has received approval for use in cats.

Cancer is an important cause of morbidity and mortality in cats [26,27,28]. In the largest study to date encompassing 51,322 cats, the incidence of cancer was reported to be 35% [26]. Among the various types of cancer, epithelial tumors emerged as the most prevalent, followed by mesenchymal and hematopoietic and lymphoreticular, with at least three quarters of all tumors being malignant in nature. Specifically, within the spectrum of malignant tumors, mammary tumors, squamous cell carcinomas (SCCs), lymphomas and fibrosarcomas (FSAs) were the most commonly reported types, and the skin and connective tissues were the most frequently affected tissues [26,27,28].

Several review articles have been published on TKs and the use of TKIs in companion animals, particularly in dogs [21,29,30]. To the best of the authors’ knowledge, however, no comparable designated review has been published on cats. The objective of this scoping review is, therefore, to systematically map the research conducted on the expression of various TKs in domestic feline neoplastic and non-neoplastic tissues, as well as the use of TKIs in domestic cats. We also aim to identify new indications for TKI use in cats, identify gaps in the knowledge and speculate on the further research warranted in this area.

## 2. Materials and Methods

The MedLine (via PubMed), CAB Abstracts and Agricola databases were searched for eligible studies with the last update on July 12th, 2023, using the following search criteria: (“tyrosine kinase inhibitor*” [tw] OR “tyrosine kinase*” [tw] OR “kit” [tw] OR “stem cell factor receptor” [tw] OR “cd117” [tw] OR “erbb” [tw] OR “her2” [tw] OR “pdgfr” [tw] OR “vegfr” [tw] OR “ron” [tw]) AND (cats[tw] OR cat[tw] OR feline[tw]). The PubMed database search also included the following medical subject headings (MeSH) terms: “Protein-Tyrosine Kinases” [Mesh] OR “Tyrosine Protein Kinase Inhibitors” [Mesh] OR “Proto-Oncogene Proteins c-kit” [Mesh] OR “Receptor, ErbB-2” [Mesh] OR “Receptor, ErbB-3” [Mesh] OR “ErbB Receptors” [Mesh] OR “Receptors, Platelet-Derived Growth Factor” [Mesh] OR “Receptor, Platelet-Derived Growth Factor beta” [Mesh] OR “Receptors, Vascular Endothelial Growth Factor” [Mesh] OR “Vascular Endothelial Growth Factor Receptor-3” [Mesh] OR “Vascular Endothelial Growth Factor Receptor-2” [Mesh] OR “Vascular Endothelial Growth Factor Receptor-1” [Mesh] OR “RON protein” [Supplementary Concept]. Studies were included if they were published in peer-reviewed journals and reported on TK expression in domestic feline neoplastic or non-neoplastic tissues or cell-lines or the use of TKIs in domestic cats. Due to the paucity of available clinical data on the subject, case reports were included. The reference lists from relevant articles and review papers were also included. In order to streamline and document the initial abstract and title screening process, all references were imported into and screened with Rayyan, a free web automation tool for systematic reviews [31]. The titles and abstracts were screened for the following exclusion criteria: (1) non-domestic cat focus of the study; (2) non-tyrosine kinases focus of the study; (3) experimental and/or molecular studies without obvious implication on feline medicine; (4) book chapters, review articles and editorials and (5) articles written in languages other than English.

The eligible studies were classified according to the following criteria: (1) tumor cell-lines, (2) non-neoplastic tissues, (3) TK expression in neoplastic tissues or serum of cats with neoplastic disease, (4) TKIs in clinical setting and (5) case reports. Tumor cell-line studies included those investigating the expression of various TKs and/or the effect of various TKIs on domestic feline tumor cell-lines. Non-neoplastic tissues encompassed investigations that sought to identify the expression of various TKs in the non-neoplastic tissues of domestic cats. The clinical studies (including case reports) on TKIs focused on assessing toxicity and response to the use of different TKIs as a part of anti-cancer, anti-pruritic or anti-inflammatory therapy.

The data extracted from a full-text review included study citation details (authors, publication year, journal title), study classification, study design, tumor type (if applicable), TKI used (if applicable), response or effect on tumor cell lines, concurrent therapy (if applicable) and toxicity (if applicable). Whenever it was available, toxicity was reported according to the VCOG-CTCAE criteria [32]. Whenever it was not available and if possible, the adverse events were graded according to the VCOG-CTCAE criteria by the authors. Furthermore, if it was available, the response in clinical studies was reported according to the RECIST [33] or WHO [34] criteria.

Due to the descriptive nature of this review, the included studies were not assessed for bias.

## 3. Results

**Figure 1:**
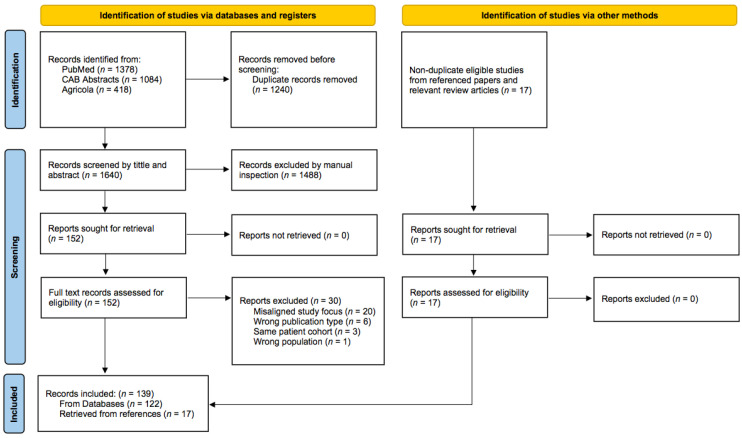
Flow diagram of the study selection process. From Page et al. [35].

Out of the initially screened 1640 articles in English, 122 met the final inclusion criteria (Figure 1). Additionally, 17 non-duplicate articles were added from reference lists of relevant articles and review papers. These publications covered a total of 25 tumor types, including mammary carcinoma (*n* = 36), mast cell tumor (MCT) of various locations (*n* = 22), SCC of various locations (*n* = 13), injection-site sarcoma (*n* = 11), gastrointestinal stromal tumor (*n* = 4), renal cell carcinoma (*n* = 4), leiomyosarcoma of the gastrointestinal tract (*n* = 3), pulmonary carcinoma (*n* = 2), iris melanoma (*n* = 2), endometrial adenocarcinoma (*n* = 2), pancreatic adenocarcinoma (*n* = 2), histiocytic diseases (*n* = 1), osteosarcoma (*n* = 1), granulosa cell tumor (*n* = 1), adrenal cortex carcinoma (*n* = 1), thyroid gland carcinoma (*n* = 1), Non-Hodgkin’s lymphoma (*n* = 1), oligodendroglioma and subependymoma (*n* = 1), colonic malignant peripheral nerve sheath tumor (*n* = 1), gemistocytic astrocytoma (*n* = 1), Merkel cell carcinoma (*n* = 1), chemodectoma (*n* = 1), tracheal adenocarcinoma (*n* = 1) and hypereosinophilic syndrome (*n* = 1). The additional studies focused on feline atopic skin syndrome (*n* = 4) and feline asthma (*n* = 1).

We also grouped the studies by the TKI that was addressed: toceranib (*n* = 13), masitinib (*n* = 8), imatinib mesylate (imatinib) (*n* = 8), oclacitinib (*n* = 6). Some studies focused on pharmacokinetics (*n* = 2) and safety/toxicity (*n* = 5).

### 3.1. Expression of Various Tyrosine Kinases in Normal Feline Tissues

The expression of TKs in normal feline tissues has been investigated through various methods, with immunohistochemistry being the predominant approach, as evidenced by a total of 28 articles documented in Table 1. So far, the expression profiles of 12 TKs have been characterized in 37 distinct normal tissues or cells. The strength of expression was not always quantified, leading to variations in the reporting of their abundance in different tissues or cells.

### 3.2. Expression and Therapeutic Targeting of Tyrosine Kinases in Feline Malignancies

The search revealed 81 publications on the expression of TK in a total of 24 different tumor types. Mammary carcinomas (*n* = 36), MCTs (*n* = 22), SCC (*n* = 13) and injection-site sarcomas (*n* = 11) are the four tumor types most commonly studied to date. A summary of expression of various tyrosine kinases, their prognostic implications and the use of TKIs in vitro and in vivo in the four most studied malignancies is provided in Table 2.

#### 3.2.1. Mast Cell Tumors

##### Tyrosine Kinase Expression in Feline Mast Cell Tumors

The expression of RTK KIT has been identified in feline MCTs at various locations, including cutaneous, splenic, intestinal and unspecified sites [56,62,63,64,65,66,67,68,69,70,71,72,73]. Unsurprisingly, the cutaneous form is the most widely studied, with various degrees of positive KIT labeling on immunohistochemistry (IHC) in 168/208 (81%; range: 55–93%) tumor samples across all reviewed studies [62,63,64,69,70,71]. The expression pattern was membranous in 87 (52%), cytoplasmic (either focal or diffuse) in 76 (45%) and reported as both in 5 (3%) samples. Cytoplasmic, and therefore dysregulated, expression was associated with a significantly worse prognosis on univariate analyses in two studies [62,63], but it was not significant in one [64]. Splenic MCTs exhibited positive KIT labelling in 67% of cases (29/43) on IHC (5 membranous and 24 cytoplasmic expression patterns) and immunocytochemistry [65,67,68,70,71,73]. However, the expression pattern did not correlate with survival [65]. In intestinal MCTs, positive KIT labeling was found in 59% of cases (22/37) (6 membranous, 11 cytoplasmic expression patterns and not reported in 5) with no association with survival [66,72].

Mutations in the *c-kit* proto-oncogene, which encodes RTK KIT, were identified in exons 6, 8, 9, 10, 11 and 18 [17,19,20,63,65,67,74,75]. The reported rate of *c-kit* mutations ranges from 56–68%. While one study found no mutations in exons 11, 12 and 17 in 10 splenic MCTs [76], a recent investigation identified mutations in exons 8, 9, 10 and 18 in a splenic MCT from a cat with splenic and cutaneous disease [20]. This study also made a significant finding by reporting, for the first time, the presence of intratumoral heterogeneity in *c-kit* mutations, suggesting that the tumor cells harbored diverse genetic alterations within the same tumor mass. It is important to note that the splenic samples were collected after a 3.5-month therapy with toceranib, raising the possibility that the TKI might have influenced the development of this heterogeneity [20]. Moreover, intriguingly, cats with multiple MCTs may exhibit distinct mutational profiles in each tumor [63]. On the other hand, no mutations have so far been identified in intestinal MCTs [66].

Thus far, mutation status does not seem to be associated with survival times, suggesting a secondary role of *c-kit* mutations in tumor behavior [65]. However, the inconsistent association between aberrant KIT protein localization and *c-kit* mutations suggests the possibility of yet undiscovered driver mutations in the *c-kit* gene [63]. For now, various functional mutations have been identified in exons 8, 9, and possibly 10, while the impact of mutations in exons 6 and 11 on KIT protein activity have not been explicitly investigated [17,19,20,63,74]. Interestingly, a suppressive mutation has been found in exon 18. Nevertheless, the precise impact of these mutations on feline MCT pathogenesis remains to be fully elucidated [20].

##### Targeting Tyrosine Kinases in Feline Mast Cell Tumors

In terms of therapeutic efficacy, imatinib, dasatinib, nilotinib and midostaurin have shown in vitro effectiveness against splenic neoplastic mast cells [67]. Toceranib [77], imatinib [17,19,78,79] and masitinib [66] have been investigated in the clinical setting. Toceranib treatment at a median dosage of 2.5 mg/kg three times per week or every other day (EOD) in 50 cats with MCTs (22 cutaneous, 10 splenic/hepatic, 17 gastrointestinal, 1 other) showed an overall clinical benefit rate of 80% (40/50), with complete response (CR) in 26% (13/50), partial response (PR) in 44% (22/50), stable disease (SD) in 24% (12/50; 7 of which were less than 10 weeks), and progressive disease (PD) in 6% (3/50) [77]. The median response duration was 32 weeks and consistent with findings in dogs [25]. Previous cytoreductive medical therapy did not negatively influence the response [77].

Imatinib (10–15 mg/kg daily) was evaluated in 13 cats (cutaneous *n* = 8 splenic/hepatic *n* = 2; cutaneous/splenic/hepatic *n* = 2; intestinal *n* = 1; together with mastocytemia *n* = 7) in one study [19] and three single reports [17,78,79]. A complete response was noted in 1 cat (8%), PR in 8 cats (62%), including at least 1 cat without a detectable *c-kit* mutation, and SD or no response in 3 cats (23%) after 2–3 weeks of therapy. In one cat (8%), CR of cutaneous tumors, but persistence of mastocytemia was observed after 5 weeks [17]. The mutation status was not reported for two cats [78,79]. Due to very varied treatment lengths, response duration was undetermined.

Masitinib was used in only two cases of intestinal MCTs with one cat surviving 538 days and the other cat being alive for at least 90 days [66]. However, detailed treatment courses were not provided. In a study focusing on feline splenic MCTs, the evaluation of TKIs was limited as only four cats received TKI therapy without specification, and they were grouped with other systemic therapies [80].

#### 3.2.2. Mammary Tumors

##### Tyrosine Kinase Expression in Feline Mammary Tumors

The expression of human epidermal growth factor receptor (EGFR, HER1, ErbB1) [81,82,83,84,85], human epidermal growth factor receptor 2 (HER2, ErbB2) [44,81,86,87,88,89,90,91,92,93,94,95,96,97,98,99,100,101], human epidermal growth factor receptor 3 (HER3, ErbB3) [83], RON kinase (Recepteur d’Origine Nantais, macrophage stimulating protein receptor, MST1R) [102,103] and vascular endothelial growth factor receptors 1 and 2 (VEGFR-1/Flt-1; VEGFR-2/KDR/Flk-1) [104,105] have been studied in feline mammary carcinoma (FMC).

Most studies focused on the expression of HER2. The investigations on HER2 expression in FMC revealed variable immunohistochemical expression rates ranging from 5.5% to 90% [44,81,86,87,88,89,90,91,92,93,95,96,97,98,99,100,101]. One study reports a higher rate of HER2 expression in benign feline mammary lesions and normal mammary tissue than in FMC [44]. The discrepancies in documented rates may be attributed to differences in antibodies, antigen retrieval techniques, scoring systems, and interpretation [44,92]. Due to these issues and in an attempt to unify reporting, several studies have also utilized in situ hybridization (ISH) in FMC [90,91,93]. Conflicting findings on the prognostic significance of HER2 overexpression in FMC have been observed [87,92,98]. Two investigations indicated a significant correlation between HER2 overexpression and shorter overall survival (OS) on univariate analysis, however, variations in the criteria used to define “overexpression” resulted in different expression rates being reported (60% or 28/47 vs. 10% or 5/51) [87,98]. Interestingly, higher HER2 expression was demonstrated in smaller tumors, leading to the proposal of its potential role in early carcinogenesis [98]. In contrast, another analysis involving 73 samples found a low expression rate of 5.5% and raised doubts about the significance of HER2 in FMC prognosis, as it found no correlation between HER2 overexpression and OS [92]. Furthermore, a separate study reported that only 16% (2/12) of HER2 overexpressing FMC showed *Her2* gene amplification [93], while another study suggested that amplification of the *Her2* gene was not relevant to its overexpression [106]. The concordance in HER2 expression between the primary tumor and metastases was not consistent in all studies [88,95]. These disparate findings highlight the complexity of HER2’s role in FMC. It remains to be determined if *Her2* amplification is a driver gene alteration in FMC carcinogenesis or only a bystander passenger mutation [90].

The results of HER2 immunohistochemical expression in two cases of inflammatory and anaplastic FMC were conflicting [107,108]. In benign or pre-invasive feline mammary lesions, the largest studies reported HER2 overexpression in 29% (17/59) and 36% (16/45) of cases, respectively [45,109].

One study compared the serum levels of HER2 extracellular domain in 60 queens with FMC and 20 healthy cats [94]. The cats with FMC had significantly higher serum HER2 levels than the healthy cats, and the cats with HER2+ FMC had higher levels than the cats with HER2- FMC. The authors suggest that serum HER2 measurement could potentially be used to predict the therapeutic response to anti-HER2 agents in cats, drawing parallels with similar suggestions in human medicine [110]. As the study also showed a false positive rate of 25–30% in healthy cats, caution is advised with interpretation. Additionally, contrasting the evidence observed in human medicine [111], HER2 levels in serum and tissue were associated with less aggressive features [94]. These contradictory findings emphasize the need for further research before the assay can be considered practical and reliable for clinical use in feline patients.

Various other TKs have been studied for their potential prognostic significance and as therapeutic targets in FMC. Notably, alongside HER2, both EGFR and HER3 expression have been identified in numerous FMC cell lines and tumor samples. Another TK of interest within the MET family, RON, and its transcript have also been shown in FMC [102,103]. Intriguingly, the presence of the transcript of its short form (sf-RON), as detected by RT-PCR, was associated with poorly differentiated tumors, a shorter disease-free interval (DFI) and a shorter survival on univariate analysis [102].

Furthermore, angiogenesis has emerged as a potential prognostic factor in invasive feline mammary tumors, as evidenced by the observed expression of VEGFR-2 and its ligand, VEGF, in the epithelial, endothelial and stromal compartments of invasive feline mammary tumors [105]. Furthermore, in cats with certain molecular subtypes of FMC (HER2+ and triple negative normal-like), serum levels of VEGFR-1, VEGFR-2 and VEGF-A were significantly elevated, implicating that these molecules may serve as non-invasive biomarkers for these specific tumor types [104].

Mutations in the *Her2* gene of FMC have also been studied [112,113,114]. One of the earliest works detected five different sequence variants in FMC samples, which was two more than in the normal mammary tissue of cats. Bioinformatics analysis identified four of those sequence variants as potential candidates for alternate splicing, possibly leading to the production of truncated protein isoforms of HER2. Additionally, an association was proposed between non-wild type haplotypes and the development of a higher number of tumors and larger tumor size [114]. Recent studies have shown that *Her2* mutations were present in the majority of FMC samples (90%, 36/40) and identified a total of 42 sequence variants. The frequency of mutations varied between four different molecular subtypes (triple-negative, HER2-positive, luminal B, luminal A), with triple-negative FMCs showing the highest degree (71.4%) of mutations and luminal A showing none. Notably, a single mutation in exon 18 was associated with a larger tumor size. As none of the identified mutations have thus far been described to induce resistance to TKIs or immunotherapy in human breast cancer (HBC) patients, it is believed the presence of these mutations should not compromise the use of TKIs in cats with FMC [112,113].

##### Targeting Tyrosine Kinases in Feline Mammary Tumors

Limited research has been conducted on the use of TKIs targeting FMC. Two studies explored the effect of four human TKIs (geftinib, AG825, GW583340 and lapatinib) on several FMC cell lines [82,83]. In one study, the dual EGFR and HER2 inhibitor GW583340 reduced cell proliferation most effectively [82], while the other study showed the dose-dependent inhibition of proliferation with both gefitinib and lapatinib [83], indicating the presence of functional RTKs in FMC. The addition of epidermal growth factor (EGF) increased the proliferation rate of FMC cell lines, suggesting a functional EGFR pathway. Furthermore, FMC cells consistently exhibited higher sensitivity to EGFR targeting compared to HER2 targeting [82].

Two additional studies investigated the antiproliferative effects of two TKIs [112] and anti-HER2 mAbs [113] in three FMC cell lines with varying levels of HER2 expression. Lapatinib demonstrated 100% cytotoxicity in all cell lines, whereas neratinib showed lower cytotoxicity rates (31–79%). A promising strategy to combat resistance development in HBC involves combining TKIs with the mTOR inhibitor rapamycin [115]. This combination demonstrated a strong synergistic antiproliferative effect in vitro [112]. In the same three feline cell lines, slightly lower dose-dependent antiproliferative effects were observed with trastuzumab (60–93%), pertuzumab (51–62%) and the antibody–drug conjugate trastuzumab-emtansine (T-DM1; 54–94%) [113]. Interestingly, combined exposures of both mAbs or mAbs and lapatinib showed a synergistic antiproliferative effect [112,113].

Bevacizumab, a recombinant humanized mAb against VEGF, suppressed tumor growth in a xenograft model of FMC but did not affect the tumor proliferation index [116]. To date, the use of TKIs in FMC has not been investigated in clinical settings. Nevertheless, one aforementioned study [83] did explore the effects of electrovaccination with heterologous feline HER2 DNA leading to the detection of high levels of IgG antibodies against human HER2 and specific feline HER2 T-cell response in some cats.

#### 3.2.3. Squamous Cell Carcinomas

##### Tyrosine Kinase Expression in Feline Squamous Cell Carcinomas

The expression of EGFR, HER2 and platelet-derived growth factor receptor α (PDGFR-α) has been studied in feline SCC [43,82,117,118,119,120]. Immunohistochemical studies have documented expression of EGFR in 69% (9/13) and 100% (67/67) of feline oral SCC [43,117] and 74% (14/19) of cutaneous [119] SCC of the head. Furthermore, in vitro studies have also shown that, similar to FMC, the addition of EGF increased the proliferation rate of oral SCC cell lines, suggesting a functional EGFR pathway [82]. The prognostic significance of EGFR expression in feline SCC, however, is currently undetermined. A smaller study identified a significant correlation between EGFR immunoexpression and a shorter DFI and OS on univariate analysis in feline cutaneous SCC of the head (*n* = 19) [119], whereas no association between EGFR expression and prognosis was found in oral (*n* = 22) SCC [118]. In contrast, a larger study (*n* = 67) indicated a trend towards better survival in feline oral SCC (FOSCC) with a higher EGFR expression score. The study discussed possible reasons for the unexpected results, including a small sample size, patient heterogeneity in terms of tumor stage and a potentially inappropriate cut-off point [117]. Furthermore, HER2 expression has been documented by Western blot, immunocytochemistry and RT-PCR in two FOSCC cell lines [82]. Finally, one study documented the immunohistochemical expression of PDGFR-α in 24/27 (89%) feline oral and cutaneous SCC, along with several angiogenic growth factors. Tumor location did not significantly impact the expression of PDGFR-α [120].

To the best of our knowledge, mutations in the genes encoding RTKs have not yet been reported in feline SCC. The sequencing of multiple cell lines revealed a wild-type *Egfr* genotype, prompting some authors to suggest that *Egfr* dysregulation in FOSCC might not be driven by mutations in the TK domain [121,122].

##### Targeting Tyrosine Kinases in Feline Squamous Cell Carcinomas

Four studies have evaluated the effects of TKIs on FOSCC cell lines [82,121,123,124]. Since silencing the *Egfr* gene by RNA interference has a profound effect on cell proliferation and colony formation, EGFR is suggested to be an important oncogenic driver, indicating EGFR targeting could have a therapeutic potential in FOSCC [121]. Geftinib, a TKI targeting EGFR, showed a reduction in cellular proliferation and migration in a laryngeal SCC cell line, but at a relatively high dose rate, which is unlikely to be achievable in vivo [82,121]. The authors also documented the development of geftinib resistance at least partly characterized by a change in cell morphology reminiscent of the endothelial to mesenchymal transition [121,124]. As with the FMC cell line, the FOSCC cell lines were also more affected by the dual EGFR and HER2 inhibitor GW583340 [82]. A recent study also demonstrated the impairment of cell proliferation by the anti-EGFR human mAb cetuximab in three feline SCC cell lines (laryngeal, gingival, lingual) [122]. Apart from that, masitinib inhibits cell proliferation by KIT inhibition but also increases COX-2 expression [123], suggesting a possible mechanism for therapy resistance and a rationale for combining masitinib with a COX-inhibitor.

In a clinical setting, the effectiveness of imatinib and toceranib was explored for feline SCC with mixed results. Imatinib showed no effect in three cats with FOSCCs [78]. On the other hand, one study retrospectively evaluated the effects of toceranib in cats with FOSCCs [125]. The therapy, involving toceranib with or without non-steroidal anti-inflammatory drugs (NSAIDs), resulted in a biological response rate of 56.5% (1 CR, 2 PR, 10 SD, 10 PD) in a group of 23 cats. Furthermore, the cats receiving toceranib exhibited a significantly longer median survival time (123 days) compared to the group of 23 cats that did not receive toceranib treatment (45 days). However, long-term survival remained disappointing, with only 3 cats (6.5%) alive at one year, all of whom received toceranib in combination with NSAIDs [125]. In a case report, a multimodal therapy including surgical resection, adjuvant radiotherapy and toceranib for anal sac SCC achieved a progression free survival of 236 days [126].

#### 3.2.4. Injection-Site Sarcomas

##### Tyrosine Kinase Expression in Feline Injection-Site Sarcomas

Histologically, most feline injection-site sarcomas (FISSs) are FSAs, but other sarcoma subtypes have also been described [127,128]. The expression of KIT, PDGFR, PDGFR-ß, EGFR, VEGFR and HER2 has been studied in FISSs [18,55,129,130,131,132,133].

Three studies investigated the immunohistochemical expression of KIT in FISSs and other soft tissue FSAs in a total of 81 cats [129,130,131]. KIT immunoreactivity was observed in 0–26% (0/14; 4/21; 12/46) of cats, with the highest expression rate found in cases that were not considered FISSs, and only 9% (4/46) of all tumors in one study showed a strong reaction [131]. Although KIT expression is not specifically disclosed for 17 FISS cases, there was no significant difference in KIT immunoreactivity between FISSs and other soft tissue FSAs. Furthermore, no significant correlation was found between KIT immunoreactivity and survival time, suggesting that factors other than KIT may contribute to the tumorigenesis of soft tissue FSAs in cats [131]. This hypothesis is supported by a study that found no KIT expression in 14 cases [129].

In the tumorigenesis of FISS, the involvement of PDGFR has been proposed [18,55,133]. A high immunohistochemical expression of PDGFR, along with EGFR and VEGFR expression, was observed in FISS [55,129,133]. Interestingly, non-injection-site sarcomas did not exhibit immunohistochemical expression of PDGFR and EGFR [133]. Moreover, PDGFR-β has also been identified in several FISS cell lines and one soft tissue FSA cell line through Western blot analysis [18,132].

Very few studies thus far have explored the genetics of FISS and feline soft tissue sarcomas in general [134]. The authors of one study used a genome-wide oligonucleotide microarray platform to detect imbalances in the DNA copy numbers of several key cancer-associated genes including *c-kit* [135]. The authors of this review are not aware of any publications regarding mutations in the genes encoding RTKs in FISS.

##### Targeting Tyrosine Kinases in Feline Injection-Site Sarcomas

In one of the earliest studies, imatinib was shown to inhibit PDGF-induced autophosphorylation of PDGFR-β in vitro. It not only reduced the viability of FISS cell-lines but also significantly inhibited the growth of FISS in a xenograft murine model compared to controls. Furthermore, imatinib was found to increase chemosensitivity to doxorubicin and (to a lesser extent) to carboplatin in vitro [18]. However, in the clinical setting, imatinib at a dosage of 1–10 mg/kg daily only achieved stable disease for an average of 2 months in four cats [78].

Similar to imatinib, masitinib has also been shown to inhibit autophosphorylation of PDGFR-β and to significantly inhibit cell proliferation in a dose-dependent manner in two FISS cell lines [132]. However, the IC_50_ values were much higher than those reported for PDGFR-expressing human cell lines [136] and these doses would likely be toxic for cats [132]. The same research group reported similar data in further studies and also found that masitinib does not enhance radiosensitivity in three FISS cell lines [137,138]. Only a single case report describes a successful adjuvant treatment of a recurrent FISS (malignant fibrous histiocytoma) with masitinib over several years [139].

To date, only one study has evaluated the use of toceranib in the treatment of FISS in 18 cats with unresectable tumors, administered at a target dosage of 3.25 mg/kg EOD [129]. The results were disappointing, with 13/14 cats showing PD after a median of 43 days (four cats were censored). Notably, one cat received palliative radiation therapy prior to toceranib and remained in SD for approximately 130 days before being euthanized due to paraparesis of unknown origin. The authors argued against the importance of the PDGF/PDGFR pathway in FISS, citing that although the majority of tumors expressed PDGFR immunohistochemically, only 35% (5/14) co-expressed both PDGFR and its ligand, PDGF [129]. However, a recent investigation identified immunoexpression of PDGFR-α and PDGFA in all 14 of the examined FISS samples [55]. This discrepancy might be attributed to the use of different PDGFR isoforms (e.g., α vs. β), as the previous study does not specify the isoform utilized.

**Table 2:** Summary of expression of various tyrosine kinases, their prognostic implications, and the use of tyrosine kinase inhibitors in vitro and in vivo in four most studied malignancies in cats.

	Location	TK Expression	Prognostic Implications of TK Expression	TKI Use In Vitro	Clinical TKI Use
**Mast cell tumor**	Cutaneous	KIT+ on IHC in 55–93% [62,63,64,69,70,71]	CTP expression associated with worse prognosis (univar. analysis) [62,63]	Imatinib, dasatinib, nilotinib, midostaurin inhibit growth and induce apoptosis in splenic MCT [67]	Toceranib (*n* = 50): CB 80% (CR 26%, PR 44%, SD 24%), mRD 32 weeks; various locations [77] Imatinib (*n* = 13): CR 8%, PR 70%, SD/no response 23%; mRD not evaluated; various locations [17,19,78,79]
	Splenic	KIT+ on IHC/ICC in 67% [65,67,68,70,71,73]	No correlation [65]		
	Intestinal	KIT+ on IHC in 59% [66,72]	No correlation [66]		
**Mammary carcinoma**		HER2+ on IHC in 5.5–90% [44,81,86,87,88,89,90,91,92,93,95,96,97,98,99,100,101]	Conflicting evidence: overexpression associated with shorter OS (univar. analysis) [87,98]; not associated with OS [92]	AG825, GW583340, lapatinib, geftinib inhibit proliferation (GW583340 most effectively among first three) [82,83] Lapatinib and neratinib induced 100% and 31–79% cytotoxicity, respectively. Addition of rapamycin had synergistic effect [112] Trastuzumab (60–93%), pertuzumab (51–62%), trastuzumab-emtansine (T-DM1; 54–94%) inhibit proliferation. Combination of both mAbs or mAbs/lapatinib had synergistic effect [113]	/
		HER2 serum levels higher than in healthy controls [94]	Higher levels associated with less aggressive features [94]		
		EGFR+ on multiple cell lines and on IHC [81,82,83,84,85]	/		
		HER3+ on two cell lines [83]	/		
		RON+ on IHC in 52% (29–68%) [102,103]	sf-RON transcript detected by RT-PCR associated with poorly differentiated tumors, shorter DFI and ST (univar. analysis) [102]		
		VEGFR-1 and VEGFR-2+ on IHC [104,105]	Serum VEGFR-1, VEGFR-2, VEGF-A levels elevated with certain FMC molecular subtypes [104]	Bevacizumab suppressed tumor growth in a xenograft model of FMC [116]	
**Squamous cell carcinoma**	Oral	EGFR+ on IHC in 69–100% [43,117,118]	No statistically significant association [117,118]	Masitinib inhibits proliferation and increases COX-2 expression [123] GW583340 inhibits proliferation [82] Geftinib inhibits proliferation (at high dose) [82,121] Cetuximab inhibits proliferation [122]	Toceranib (*n* = 23): CR 4%, PR 9%, SD 43%, PD 43%; increased mST compared to control (123 vs. 45 days) [125] Imatinib (*n* = 3): no effect [78]
		HER2+ in two cell lines [82]	/		
	Cutaneous (head)	EGFR+ on IHC in 74% [119]	Expression assoc. with worse prognosis (univar. analysis) [119]	/	/
	Oral and cutaneous	PDGFR-α+ on IHC in 89% [120]	/		
**Injection-site sarcoma**		KIT+ on IHC in 0–26% [129,130,131]	No correlation [131]	Imatinib inhibits PDGFR-β to reduce viability of FISS cells and to significantly inhibit the growth of FISS in a xenograft murine model. Increased chemosensitivity to DOX and CARBO [18]	Toceranib (*n* = 14): SD 7, PD 93% [129] Imatinib (*n* = 2): SD for average RD of 2 months [78]
		PDGFR+ on IHC in 100%; only 35% both PDGF/PDGFR+ [129]; PDGFR-α/PDGFA+ on IHC in 100% [55] PDGFR-β+ by Western blot in vitro [18,132]	/		
		VEGFR+ on IHC in 93% [129]	/	Masitinib inhibits proliferation (at high dose) [132,137,138]	
		EGFR: “consistent strong staining” [133]	/		

IHC = immunohistochemistry; ICC = immunocytochemistry; CTP = cytoplasmic; CB = clinical benefit; CR = complete remission; PR = partial remission; SD = stable disease; mRD = median response duration; mST = median survival time; DOX = doxorubicin; CARBO = carboplatin; DFI = disease free interval; univar. = univariate.

#### 3.2.5. Gastrointestinal Stromal Tumors

Gastrointestinal stromal tumors (GISTs) are extremely rare in cats with few case reports published [140,141,142,143]. Similar to people [144] and dogs [14,145], a deletion in exon 11 of the *c-kit* gene and immunoreactivity for KIT have been identified in the cat [56,140,143], implying a similar role in the tumorigenesis of feline GISTs.

A single case report describes the medical management of an unresectable gastric GIST with toceranib for over 18 months with the cat’s disease remaining stable (reduction in maximal tumor diameter of 29.5%) [141]. In another case report, imatinib and toceranib also achieved SD in a small intestinal GIST, but both treatments were discontinued due to adverse effects (hyperbilirubinemia, which resolved after drug discontinuation). No gain-of-function mutations in the *c-kit* and *PDGFRA* genes were identified in this cat [142].

#### 3.2.6. Other Malignancies

Apart from the above-mentioned tumors, TK expression and/or TKI treatment has been investigated in feline pulmonary carcinomas [146,147], endometrial adenocarcinoma [46], feline histiocytic disorders [54], renal cell carcinoma [58,59,148,149], iris melanomas [150], granulosa cell tumors [56], adrenal cortex carcinoma [56], thyroid gland carcinoma [56], Non-Hodgkin’s lymphoma [56], leiomyosarcoma of the gastrointestinal tract [140,151,152], osteosarcoma [57], oligodendrogliomas and subependymomas [153], colonic malignant peripheral nerve sheath tumors [154], gemistocytic astrocytoma [155], Merkel cell carcinoma [156], pancreatic adenocarcinoma [157,158], chemodectoma [159], tracheal adenocarcinoma [160] and hypereosinophilic syndrome [161].

One recent study investigated the expression of HER2 by IHC and *Her2* gene amplification by ISH in 13 feline pulmonary carcinomas [146]. HER2 overexpression and amplification was identified in 15% and 27% of cases, respectively, and overexpression always corresponded to gene amplification. HER2 overexpression, amplifications and mutations are well characterized in human non-small-cell lung carcinomas (NSCLC) with possible therapeutic implications [162,163], which could show potential in cats as well. Similarly, mutant EGFR protein expression was detected in 5/24 (21%) feline pulmonary carcinomas, but its prognostic effect could not be evaluated [147].

HER2 immunoexpression was also demonstrated in 20/34 (59%) feline endometrial adenocarcinomas (FEA). However, no correlation was found between HER2 expression and various pathological features, such as nuclear atypia, mitotic number, and myometrium, serosa and vascular invasion. As a result, the impact of HER2 on progression and prognosis of FEA has been called into question [46]. On the other hand, the immunoexpression of HER2 was also reported in the hyperplastic endometrial polyps of two cats with a cystic endometrial hyperplasia-pyometra complex, suggesting its potential involvement in the pathogenesis of endometrial hyperplasia [47,164].

One study investigated PDGFR-β and KIT expression by IHC in 15 cases of various feline histiocytic disorders (five feline progressive histiocytoses, eight histiocytic sarcomas and two hemophagocytic histiocytic sarcomas) [54]. PDGFR-β expression was observed in 87% of cases, with 66% exhibiting strong positive staining, while no KIT staining was detected in any of the samples. Almost all cats surviving longer than 300 days from diagnosis showed high PDGFR-β expression, which the authors attributed to possibly higher tumor differentiation. Two cats with histiocytic sarcoma and progressive histiocytosis achieved CR with masitinib for a duration of 110 and 223 days, respectively. The second cat was transitioned to toceranib and remained alive 1800 days later despite PD. However, due to the limited number of cats receiving TKIs, the correlation between PDGFR-β expression and response to TKIs could not be evaluated. The authors propose PDGFR-β as a possible marker of differentiation with a prognostic potential [54]. Unsurprisingly, a case of ocular histiocytic sarcoma in a cat was also immunonegative for KIT [165].

Two studies [58,59], a case series [148] and a case report [149] investigated the immunohistochemical profiles of renal cell carcinomas. Diffuse cytoplasmic immunoexpression of KIT was documented in 23/37 (62%, range 0–92%) cases. The discrepancies in expression rates may be due to different antigen retrieval methods [58]. Furthermore, VEGFR-2 immunoexpression was shown in 4/4, while its ligand, VEGF, was positive in 2/4 cases, potentially reflecting variable activity among different tumors [148].

Various studies have investigated the expression of KIT and its potential therapeutic implications in different feline malignancies [56,57,150,156,166]. Immunofluorescence analysis revealed the overexpression of KIT in 57 feline diffuse iris melanomas, suggesting a possible role in the tumorigenesis of these tumors and a potential therapeutic target [150]. However, no mutations in the *c-kit* genetic regions were identified in 11 iris melanomas and one conjunctival melanoma [166].

In contrast, a weak immunohistochemical expression of KIT was demonstrated in a single case of a granulosa cell tumor [56], while no KIT expression was identified in a gastrointestinal leiomyosarcoma (*n* = 1), endometrial adenocarcinoma (*n* = 1), adrenal cortex carcinoma (*n* = 1), thyroid gland carcinoma (*n* = 1) and Non-Hodgkin’s lymphoma (*n* = 2). Similarly, KIT immunoexpression was not observed in two further case reports of a duodenal leiomyosarcoma [151] and an esophageal angioleiomyosarcoma [152].

Furthermore, in four cases of feline osteosarcomas (OSA), no immunohistochemical expression of KIT was detected. This contradicts the results from canine OSA and implies a different role of KIT in the tumorigenesis of OSA in both species [57].

In a very recent study, feline oligodendrogliomas and subependymomas were immunopositive for PDGFR-α [153]. Moreover, a rare case of a colonic malignant peripheral nerve sheath tumor with hepatic metastasis showed faint immunoreactivity for KIT, leading to the exclusion of GIST based on positive staining for S-100 and the unconvincing nature of KIT staining [154]. Additionally, in another rare case report, a spinal cord gemistocytic astrocytoma was immunonegative for EGFR [155]. In a case series of feline (and canine) Merkel cell carcinomas, all three feline specimens reacted positive for KIT, but the lymphoid origin could not be demonstrated [156].

In the context of therapeutic interventions, two case reports documented the treatment of cats with pancreatic adenocarcinomas using adjuvant or single-agent toceranib, resulting in reported survival times of over 1436 and 792 days, respectively [157,158]. Similarly, a single case report describes a yearlong adjuvant treatment with toceranib following the surgical excision of a caval chemodectoma, leading to a survival time of 2.5 years [159]. In another case report, a cat received toceranib over four months as part of a multimodal therapy against tracheal adenocarcinoma, resulting in a survival time of 755 days [160]. Finally, imatinib was used to treat one cat with hypereosinophilic syndrome at a prescribed dosage of 9.6 mg/kg daily. After an initial clinical improvement and normalization of peripheral eosinophil count, the therapy was discontinued eight weeks later due to the development of minimal change glomerulopathy [161].

### 3.3. Non-Neoplastic Diseases

Masitinib (50 mg daily) was shown to reduce eosinophil count and total protein level in the bronchoalveolar lavage fluid of 12 cats with experimentally induced asthma compared to placebo controls [167]. The results of the pulmonary mechanic testing also indicated enhanced respiratory compliance in the treated cats. Considering these findings, the authors proposed that cats could serve as a model for human medicine [167]. Indeed, masitinib has shown promise in humans with severe asthma [168].

The effectiveness of oclacitinib against feline allergic dermatitis has been evaluated in several studies and at least 53 cats [169,170,171]. Mean dosages of 1 mg/kg once or twice daily reduced the validated Scoring Feline Allergic Dermatitis (SCORFAD) [172] and Pruritus Visual Analogue Scale (PVAS) [173] scores ≥50% in 60–88% and 61–70% of cats, respectively [169,170]. The effects were less pronounced but still visible with 0.5 mg/kg twice daily dosages [171]. When compared to methylprednisolone, the latter performed better, but oclacitinib could present a useful alternative when glucocorticoids are contraindicated [169].

### 3.4. Pharmacokinetics, Safety and Toxicity of Commonly Used Tyrosine Kinase Inhibitors in Cats

#### 3.4.1. Toceranib

Toceranib is a potent and selective inhibitor of several members of the split-kinase domain family of RTKs, including VEGFR, PDGFR, Flt-3 and KIT [174]. The authors of this review found no studies evaluating the pharmacokinetics of toceranib in cats. Excluding single case reports, several retrospective [77,125,175,176,177] and prospective [129] studies have evaluated the safety of toceranib in a total of 195 cats. Toceranib was mostly prescribed at a dosage of 2.5–2.78 mg/kg three times per week or EOD, and it was generally well tolerated [77,125,175,176,177]. Only a prospective study utilized a 3.25 mg/kg EOD dosing schedule and reported good tolerability and generally mild and temporary side effects [129]. The most commonly reported side effects in 91 out of 195 cats (47%) were mild (VCOG grade 1 and 2) gastrointestinal upset, characterized by symptoms such as anorexia, vomiting, diarrhea or a combination thereof. Notably, as some studies also evaluated cats with oral SCCs [125,176,177] and visceral and gastrointestinal MCTs [77,176], some of these adverse events may also be attributed to the primary disease. Commonly reported were lethargy, mild hematological (neutropenia, lymphopenia, anemia, thrombocytopenia) and biochemical (increased alanine aminotransferase [ALT] and azotemia) abnormalities. VCOG grade 3 and 4 adverse events were observed in 22 out of 195 cats (11%), with 12 of these cases (55%) showing an increased ALT. Among the 12 cats with grade 3 and 4 ALT elevation, at least 7 received a dosage of 3.08 mg/kg toceranib three times per week or more. In dogs, the clearance of toceranib is primarily hepatic [178] and the same may be true for cats. It has been suggested that the higher degree of liver toxicity in cats may be due to their impaired glucoronidation mechanism [176].

One study reports two cases of grade 5 toxicities (azotemia and anemia) resulting in death in cats with visceral MCTs. However, the authors suspected neither of these events were related to toceranib administration [77]. One cat had received toceranib for 149 weeks prior to developing azotemia [77].

While several studies report occasional cases of long-term administration (100 weeks or more), none have specifically evaluated the long-term safety of toceranib in cats [77,175,177].

Urinalyses with urinary protein:creatinine ratio (UPC) were assessed in 30 cats [125,129]. Although grade 1 and 2 proteinuria measured by urine dipstick were reported in 12/18 cats in one study, UPC was mildly elevated (>0.8) in only one case [129]. In another study, none of the 12 cats with available data had significant proteinuria (UPC > 0.4) [125].

A single case report documents the development of hair coat hypopigmentation after 14 months of toceranib treatment [157]. Similarly, skin depigmentation was also reported in dogs [179].

#### 3.4.2. Masitinib

Masitinib most effectively and selectively targets KIT TK and, to a lesser extent, PDGFR, Lyn and fibroblast growth factor receptor 3 (FGFR3) [136]. In cats, the bioavailability of masitinib after oral administration is lower (60% vs. 80%) and it has a shorter half-life (3–5 h vs. 10–20 h) when compared to dogs [180]. The peak concentration is reached after two hours (~1 μM) and is also lower than in dogs, but above the reported IC_50_ of human and murine KIT [24,180].

Tolerability has been evaluated on 26 cats in two studies [167,181] and in occasional case reports [139]. The dosing schedule of 50 mg/cat daily or EOD was mostly well tolerated. The most common adverse events reported are vomiting (10/20 cats and four events), diarrhea (3/20 cats and one event), neutropenia (3/26 cats) and proteinuria (8/26 cats). Aside from proteinuria, the adverse events were VCOG grade 1 or 2 and self-limiting. Proteinuria was noted in 30% of the cats (VCOG grade 3 in two [181] and “moderate to severe” in 6/6 [167]). However, none of the cats on the EOD schedule developed proteinuria and, importantly, proteinuria resolved spontaneously after discontinuation of masitinib in all cases. As the cats in these studies were either healthy (*n* = 20) [181] or had experimentally induced asthma (*n* = 6) [167], the adverse effects are likely associated to masitinib. Serum creatinine increased and serum albumin decreased significantly from baseline during the study, but remained within the reference range [181]. A self-limiting increase in ALT was reported for one cat. As both studies only continued for 4 weeks, the long-term effects and toxicity of masitinib in cats are not well documented.

In a single case report, a cat tolerated a dosage of 50 mg (12.5 mg/kg) EOD for over a year without any clinical side effects apart from mild nausea and vomiting in the first month of treatment [139]. However, no clinicopathological data were provided.

#### 3.4.3. Imatinib

Imatinib was developed as an inhibitor of the BCR-ABL TK and targets BCR-ABL, KIT, PDGFR, colony stimulating factor 1 receptor, ABL1, ABL2, discoidin domain receptor 1/2 and lymphocyte-specific protein tyrosine kinase [182,183]. No studies evaluating the pharmacokinetics of imatinib in cats are known to the authors of this review.

Tolerability was reported in 26 cats in one dose escalating study [78], one prospective study [19] and multiple case reports [17,79,161,184]. The dosing range was wide (1–15 mg/kg daily) and the adverse events inconsistently reported. The median time of treatment in the two larger studies [19,78] was 9 weeks. The most commonly used dosage was 10 mg/kg daily. Imatinib was generally well-tolerated with only occasional and mild adverse events (VCOG grade 1 vomiting and lethargy) [78], as well as a single case of mildly to moderately increased AST and ALT [19]. One cat developed a grade 4 ALT increase two weeks after starting therapy at 1 mg/kg daily, but the causal relationship to imatinib is uncertain as the cat had received previous therapy including multiple doses of lomustine, gemcitabine and carboplatin [78]. Similar to cases reported in dogs with masitinib [185,186], one feline case report documents minimal change glomerulopathy development within two months of initiating a 9.6 mg/kg daily therapy with imatinib [161]. Proteinuria resolved partially after imatinib was discontinued. Finally, it is worth noting that cardiotoxicity, a well-known phenomenon with imatinib use in human medicine [187,188], has not (yet) been reported in cats.

#### 3.4.4. Oclacitinib

Oclacitinib is a selective inhibitor of Janus kinase 1, licensed for the treatment of clinical signs of canine atopic dermatitis and allergic pruritus in dogs [189]. Pharmacokinetics [190] and the safety of oclacitinib have been evaluated in several prospective [169,170,171,191] studies in 6 and 80 cats, respectively. After oral administration of 1 mg/kg, the drug showed high and rapid absorption and elimination, reaching a mean bioavailability of 87% within 35 min and a half-life of 2.3 h [190], similar to the pharmacokinetics observed in dogs [192].

The majority of cats tolerated mean doses of 0.5–2 mg/kg twice daily over four weeks very well. The observed adverse events were occasional mild, but not VCOG-graded, vomiting and soft stools, while hematological and biochemical abnormalities included one case of grade 3 anemia, two cases of grade 4 ALT increases and four cases of grade 1 and 2 azotemia [169,170,191]. The anemia resolved after oclacitinib was discontinued [170]. Furthermore, there are anecdotal reports of peripheral lymphadenopathy [191].

One report documents a case of fatal disseminated toxoplasmosis after five months of oclacitinib treatment. However, since the cat was also positive for feline immunodeficiency virus, the contribution of oclacitinib to the susceptibility of toxoplasmosis remained undetermined [193].

A summary of the most common adverse events of all four described TKIs is presented in Table 3.

## 4. Discussion

The aim of this review was to provide a comprehensive overview of the expression of various TKs in feline neoplastic and non-neoplastic tissues, as well as the use of TKIs in domestic cats. In spite of the considerable number of studies identified in our search, our review shows that our current knowledge of TKs and TKIs in cats is still very limited. Our review unveils the current gaps in knowledge and controversies surrounding TKs and TKIs in feline oncology, emphasizing the need for further investigations in this field. In our analysis of TK expression in normal feline tissues, we found that several studies have contributed to our understanding of TKs’ normal functioning and signaling pathways in cellular processes. By comparing TK expression levels between normal and diseased tissues, researchers can identify potential biomarkers that indicate the presence or progression of specific diseases. Studying TK expression in normal tissues helps identify which TKs are commonly expressed and which ones are unique or overexpressed in disease conditions. This information aids in target identification for developing targeted therapies that specifically modulate the activity of dysregulated TKs, such as TKIs. The knowledge of TK expression in normal tissues also helps researchers in identifying potential off-target effects of TKIs and assessing their safety profiles. Our review has shown that while the expression of KIT in normal feline tissues is well-characterized [56], the expression of PDGFR, another TK targeted by commercially available TKI, and HER2 (ErbB2) is currently less well-defined. Further studies focusing on the expression of PDGFR and HER2 (ErbB2) in normal feline tissues would, therefore, be valuable for elucidating their role in disease conditions and the potential off-target effects of TKIs.

In our exploration of TK expression in feline malignancies, we found that while TKI expression has been investigated in 21 different tumor types, nearly half of these records are either single cases or merely report the lack of a single TK expression (KIT or EGFR). Mammary carcinomas, MCTs, SCCs, and FISSs were the most commonly studied tumor types. While some progress has been made in understanding the expression and clinical implications of TKs in these tumor types, significant knowledge gaps still exist.

For instance, in MCTs, the expression of the RTK KIT and its association with prognosis has been investigated. The RTK KIT protein is expressed in feline mast cell tumors in different locations, with cytoplasmic expression linked to a worse prognosis in the cutaneous form [62,63]. It is worth noting that the prognostic significance was only documented in univariate analysis. The further research can investigate the underlying mechanisms and the specific impact of cytoplasmic expression on disease progression and patient outcomes. This could involve larger-scale studies with a diverse population of MCT cases to validate the association between cytoplasmic KIT expression and prognosis. Mutations in the *c-kit* gene are present in a significant percentage of feline MCTs [17,19,20,63,65,67,74], but their association with survival has not yet been proven [65]. The future research could focus on understanding the specific types and frequencies of *c-kit* mutations in feline MCTs and their correlation with clinical outcomes. This can provide valuable insights into the prognostic significance of different *c-kit* mutations and their potential as predictive markers for treatment response and survival in feline MCT patients. In dogs with MCTs, recent studies have revealed that mutations in different exons can be associated with a difference in prognosis [194,195].

Toceranib, imatinib and, to a lesser degree, masitinib have shown efficacy in treating MCTs [17,19,66,77,78,79], with toceranib demonstrating an overall clinical benefit rate of 80% in cats [77]. The further research can compare the efficacy of these TKIs in larger cohorts of MCT patients, evaluating response rates, progression-free survival and long-term outcomes. Additionally, investigating the factors associated with treatment resistance or relapse can help optimize treatment strategies and identify potential biomarkers for predicting the response to specific TKIs. From canine oncology, it is known that MCTs can exhibit heterogeneity and variable responses to TKIs [24,25,196,197,198], and exploring combination therapies involving TKIs and other treatment modalities (such as conventional chemotherapy, surgery or radiation therapy) can be valuable. Studies in dogs have shown promising response rates when combining toceranib with conventional cytotoxic agents such as vinblastine [199,200,201]. Conversely, the combination with lomustine proved disappointing due to high toxicity [202]. Investigating the optimal sequencing of TKIs with other treatments and evaluating their synergistic effects can help improve treatment outcomes and guide clinical decision-making.

The expression of various RTKs has been most extensively studied in FMC [44,81,82,83,84,85,86,87,88,89,90,91,92,93,94,95,96,97,98,99,100,101,102,103,104,105,107,108]. Feline mammary carcinomas share similarities with HBC and have been proposed as a model for HBC [44,81,86,89,91,93,96]. While the negative prognostic effect of HER2 overexpression is well documented in HBC [203], based on the current state of the research, controversies surrounding HER2 expression and its correlation with prognosis in cats were observed. Due to the discrepancies in reported rates of HER2 expression in FMC, further research is needed to establish standardized methods as used in HBC [204] for assessing HER2 expression, including the selection of antibodies, antigen retrieval techniques, scoring systems and interpretation. Furthermore, more research is required to validate the potential use of serum HER2 and sf-RON measurements as a predictor of prognosis and therapeutic response in cats and to investigate the underlying biological mechanisms.

While TKIs have shown efficacy in inhibiting FMC cell proliferation in vitro, their clinical use in FMC has not been investigated. The limited research conducted in vitro showed promising results as five TKIs have demonstrated various efficacy in FMC cell lines, indicating the presence of functional RTKs in these tumors [82,83,112]. The dual EGFR and HER2 inhibitor GW583340 exhibited the most effective reduction in cell proliferation in FMC cell lines [82], but also, gefitinib, lapatinib and, to a lesser extent, neratinib demonstrated cytotoxic potential in FMC cell lines [83,112]. This documents the potential of TKIs in FMC and highlights the need for more comprehensive studies to evaluate the efficacy of different TKIs. Since the authors did not find any studies on the use of TKIs in the clinical setting, the future research should focus on evaluating the safety, tolerability and therapeutic outcomes of the above-mentioned TKIs and, potentially, toceranib in FMC patients. This is especially important since the role of adjuvant conventional cytotoxic chemotherapy in FMC is debatable [205,206]. Another treatment option to explore would be the use of mAbs. Although humanized mAbs have shown efficacy in vitro and in a xenograft model of FMC [113,116] and a 92% homology between feline VEGF and human VEGF-A, and also, the immunoreactivity of bevacizumab with feline VEGF has been demonstrated [207], simply administering humanized mAbs to veterinary patients is unlikely to be successful. Possible complications include inactivation of the mAb by the recipient’s immune system or development of (severe) adverse reactions [208,209]. The production of a caninized mAb against canine EGFR and its efficacy against two canine mammary cell lines has been demonstrated [210], indicating that such a treatment could also be feasible in cats. However, the costs of speciation and production of mAbs present a challenge even in human medicine, let alone veterinary medicine [208]. Currently, while a felinized anti-nerve growth factor mAb (frunevetmab) is registered for the treatment of osteoarthritic pain in cats [211], no felinized anti-tumor mAbs are commercially available.

Furthermore, our analysis of feline oral and cutaneous SCCs revealed limited research on TK expression in these tumor types [43,82,117,118,119,120]. Similarly to FMC, FOSCC was proposed as a spontaneous model for human head and neck SCC [212]. Currently, the prognostic significance of EGFR expression in feline SCC remains undetermined [117,118,119]. Further studies with larger sample sizes and standardized methodologies are needed to evaluate the correlation between EGFR expression and prognosis in feline SCC. Given the potential therapeutic relevance of TKIs in SCCs and the current lack of effective conventional chemotherapeutic agents [213], further investigations into the expression profiles of TKs, such as EGFR and VEGFR, are warranted. While some studies have demonstrated the inhibitory effects of EGFR and HER2 inhibitors on feline SCC cell lines [82], further research is needed to evaluate the efficacy of these inhibitors in in vivo models and clinical setting. The effectiveness of imatinib and toceranib in feline SCC remains inconclusive [78,125]. Further studies with larger sample sizes and controlled clinical trials are necessary to evaluate the efficacy and long-term survival outcomes of these therapies in feline SCC. Investigating the effects of dual EGFR and HER2 inhibitors, such as GW583340 [214], could provide valuable insights into novel treatment approaches for feline SCC. Similarly, mAbs, such as cetuximab, could prove invaluable in the treatment of FOSCC, supported by the recent evidence of its in vitro activity [122]. The absence of known mutations in the feline *Egfr* TK domain suggests that cetuximab could be effective in cats, as these mutations are suspected to contribute to the reduced efficacy of this drug for human head and neck SCC patients [122,215].

In the case of FISS, the expression and clinical implications of TKs, including KIT, PDGFR, EGFR and VEGFR, have been investigated [18,55,129,130,131,132,133]. However, further studies are needed to determine the prevalence and clinical implications of these TKs in FISS. Assessing the correlation between TK expression and specific tumor subtypes within FISS, as well as evaluating the efficacy of TKIs in larger cohorts of FISS patients, would provide a more comprehensive understanding of TK expression patterns and treatment options in FISS. Studies evaluating the efficacy of TK inhibitors, such as imatinib, masitinib and toceranib, in FISS treatment have yielded mixed results [18,78,129,132,137,138,139]. Even though imatinib has shown some promise in vitro [18], only one study has evaluated a TKI (toceranib) specifically in FISS in a clinical setting with disappointing results [129]. In comparison, in human medicine, TKIs have not yet entered the mainstream of sarcoma treatment, which might also be due to the heterogeneity of these tumors [216]. Further research is needed to understand the factors contributing to variable responses and to optimize the use of these inhibitors. Assessing the effectiveness of TK inhibitors in different FISS subtypes and exploring potential combination therapies could improve treatment outcomes. Investigating the mechanisms of resistance to TKIs, particularly imatinib and masitinib, and exploring the significance of the PDGF/PDGFR pathway in FISS tumorigenesis are interesting research directions. The limited long-term survival outcomes in cats with FISS, even with the use of TKIs [129,217,218], highlight the need for improved treatment strategies. Conducting controlled clinical trials with larger sample sizes and longer follow-up periods can provide more accurate assessments of the efficacy and long-term survival benefits of different treatment modalities in FISS.

As for the other tumor types investigated, the expression of HER2 in feline pulmonary carcinomas [146,147] suggests the possibility of targeting HER2 as a therapeutic approach. Further research is needed to investigate the effectiveness of HER2-targeted therapies in feline pulmonary carcinomas with HER2 alterations, which can provide valuable insights into potential treatment options for cats. Additionally, studying the correlation between PDGFR-β expression and the response to TKIs in feline histiocytic disorders can determine the prognostic value of PDGFR-β and its potential as a therapeutic target. Further exploration is also needed to understand the role of KIT overexpression in feline renal cell carcinomas [58,59,148] and diffuse iris melanomas [150] and to evaluate the efficacy of KIT inhibitors as potential treatments for these specific types of tumors.

Further research is needed to expand our understanding of TK expression across various tumor types in cats. By exploring TK expression in a wider range of feline tumors, the potential targets for TKI therapy can be identified, and the efficacy of TKIs in different tumor types can be evaluated. Since certain feline tumors serve as valuable spontaneous models for human cancers [44,81,86,89,91,93,96,212], investigating TKI therapy in these models can enhance our knowledge of treating corresponding human cancer types, ultimately benefiting both feline and human oncology.

In recent years, the field of oncology has undergone a profound transformation, with the integration of genomic and mutation data playing a pivotal role in guiding therapeutic strategies [219,220]. While our review has comprehensively examined the landscape of RTK expression in feline cancer, it is essential to underscore the growing relevance of genomic insights. As a recent review on feline oncogenomics unravels the current state of knowledge, it becomes increasingly evident that further research is imperative to better understand the genetic basics of feline cancer [134]. In human oncology, the targeted use of RTK inhibitors has become linked to a better understanding of genetic mutations within tumors [219,220,221]. Mirroring these advances in human medicine, a comprehensive understanding of feline cancer genomes holds the promise of identifying novel targets for future therapeutic development. The recent publication of a high-quality cat genome reference lays the groundwork for future research in this domain [222].

In the clinical setting, there are currently no licensed TKIs in cats and no prospective, randomized placebo-controlled clinical trials have been conducted thus far on the use of TKIs in feline oncology. To date, four tyrosine kinase inhibitors have been used in the clinical setting for cats: toceranib, masitinib, imatinib and oclacitinib [17,77,78,129,139,167,169,170,175,176,177,181]. In cats, the use of TKIs was investigated most thoroughly for feline MCT. However, fewer than 70 feline MCTs treated with TKIs have been reported to date, and this includes case reports [17,19,66,77,78,79]. This is in contrast to dogs where numerous clinical studies have been conducted on the use of TKIs in dogs with cancer over the course of the last 20 years, and 2 TKIs have been licensed against canine MCTs [24,25].

The current state of research on the pharmacokinetics, safety and toxicity of these TKIs in cats is limited [19,77,78,125,129,167,170,171,172,175,176,177,181,191]. The available pharmacokinetic data suggest that masitinib has lower bioavailability and a shorter half-life in cats compared to dogs [180]. On the other hand, oclacitinib appears to have high and rapid absorption and elimination in cats [190]. However, comprehensive pharmacokinetic studies on these drugs in cats are lacking. Additionally, there are no studies specifically evaluating the pharmacokinetics of toceranib and imatinib in cats. The future research should focus on investigating the absorption, distribution, metabolism and elimination of these TKIs in cats. This research would help better understand factors that may affect drug absorption, such as food or concurrent medications. The distribution of TKIs in cats, including their binding to plasma proteins and potential accumulation in specific tissues, also needs further investigation. It is currently unclear how age, renal or hepatic impairment or interactions with other drugs may impact the elimination of TKIs from the body. Based on the limited available research on dosage and safety of these TKIs in cats, the currently used dosages of toceranib (2.5–2.78 mg/kg three times per week or EOD) and masitinib (50 mg/cat daily or EOD) appear to be well tolerated by most cats. Severe or irreversible adverse effects are rare at these dosages. However, the safety of long-term TKI use in cats has not been studied, and no phase 1 clinical trials have been conducted to determine the biologically effective dose (BED) or the optimal dosage regimen to achieve desired drug levels in the body. Additionally, information on potential drug–drug interactions is missing, which is crucial considering that many cats with cancer are older animals receiving concurrent medications for other medical conditions.

Similar to dogs [24,25,198], the most common adverse effects of TKIs in cats are gastrointestinal, but some important differences exist between the drugs. While ALT increase is common with toceranib and speculated to be linked to impaired hepatic glucoronidation process in cats [176], it is less commonly reported with other TKIs [19,77,129,170]. Regarding toceranib, cautious administration is, therefore, advisable in cats with preexisting hepatic conditions due to the potential risks. Masitinib, on the other hand, induced clinically significant proteinuria in nearly one third of all cats, surpassing the reported 18% occurrence in dogs [223]. Despite cats being seemingly more prone to masitinib induced proteinuria than dogs, the authors of this review have not come across any reports of nephrotic syndrome or minimal change glomerulopathy, as seen in dogs [185,186]. It is possible that the limited duration of masitinib administration in cats, mostly restricted to four weeks [167,181], could have contributed to this discrepancy. Notably, proteinuria only developed with daily dosing, suggesting that an EOD dosing schedule might provide a safer alternative for cats.

Although all four clinically used TKIs (toceranib, masitinib, imatinib, oclacitinib) are seemingly well tolerated in cats, no study to date has evaluated the long-term effects of these drugs, and the data remain limited to single cases [77,139,175,177]. As long-term side effects of chronic TKI use are well known in people [224,225], these risks cannot be excluded in cats and should be investigated. The safety profiles of TKIs, particularly with prolonged use, remain uncertain, and the risk of developing chronic side effects cannot be excluded.

Given the current lack of robust clinical evidence to support the efficacy and safety of TKIs in cats, the published dose recommendations should be interpreted with caution and their accuracy and appropriateness need to be confirmed through rigorous pharmacokinetic studies.

Our scoping review has several limitations that should be acknowledged. Although we implemented a broad and systematic search strategy proposed by the rare available literature on conducting systematic searches in veterinary literature [226], it is possible that some relevant studies may have been missed. Despite our efforts to be comprehensive, the vast and ever-growing body of literature in this field makes it challenging to capture every relevant publication. Additionally, the inclusion criteria and search terms used in our review may have inadvertently excluded certain studies that could have contributed valuable insights. Therefore, it is important to consider that our findings may not represent the entirety of the available current evidence. For the sake of maintaining the integrity of our study, we have chosen to exclude abstracts and non-peer-reviewed literature from our analysis. Consequently, some data that may have been available in those sources have not been incorporated into our research. By focusing solely on peer-reviewed publications, we aimed to ensure the reliability and credibility of the information presented in our study. Furthermore, we acknowledge that some potentially important gray literature might have been missed and, therefore, was not included in this review.

Furthermore, the limited availability of data on the use of TKIs in cats resulted in a relatively small pool of evidence to draw conclusions from. This is mostly due to the relatively small number of studies included in the clinical setting in general and the absence of prospective, randomized placebo-controlled clinical trials on the use of TKIs in feline oncology in particular. As a result, we have decided to include single case reports, which do not provide a high level of evidence (Oxford clinical evidence based medicine, OCEBM level 4). Finally, as the reporting of responses and adverse events was not uniform and consistent in all included studies, direct comparison was not always possible.

In spite of these limitations, our scoping review provides a valuable overview of the current state of research in the field. It highlights existing studies and their findings, shedding light on the knowledge gaps and areas that warrant further investigation. By recognizing these limitations, we can encourage future researchers to build upon our work and address the gaps in the literature.

## 5. Conclusions

In conclusion, while TKIs have become the standard of care in various human and canine malignancies, their use in cats remains underexplored, and no TKI is currently licensed for use in feline oncology. Although some in vitro evidence regarding the efficacy of various TKIs in feline malignancies exists, the translation of these data into clinical setting is limited, resulting in sparse information on the dosage, safety and efficacy of most TKIs in cats. The most robust clinical data focus on the use of toceranib in feline MCTs, with a small sample size of less than 70 cats. Interestingly, certain feline tumors have been proposed as spontaneous models for some human malignancies, presenting an opportunity to explore new therapeutic approaches involving human TKIs or the development of felinized mAbs.

The future investigations could concentrate on the expression of various TKs in different feline malignancies involving a larger number of cats to better predict their prognostic value and the potential for TKI use. In addition, gaining a deeper understanding of the molecular drivers of feline cancers could catalyze the evolution of new targeted therapeutics for cats. While the use of already available human TKIs holds promise in treating FMCs and FOSCCs, it is crucial to first evaluate their pharmacokinetic and safety profiles in feline patients. As cats seem to tolerate the already available veterinary TKIs, such as toceranib, well, their use could hold potential in a broader spectrum of malignancies beyond those already described. While our scoping review highlights some fundamental knowledge gaps related to TK expression and the use of TKIs in a variety of feline malignancies, we believe it also lays the important groundwork for further research in this area.

## Figures and Tables

**Table 1:** Expression of various tyrosine kinases in normal feline tissues.

Tyrosine Kinase	Tissue	Method
trkA	Dorsal pontine tegmentum [36]	IHC
	Extraocular motor nuclei [37]	IHC, ICC
trkB	Extraocular motor nuclei [37] Olfactory bulb [38] Visual cortex [39]	IHC, ICC ISH IHC
	Lateral geniculate nucleus [40]	IHC
trkC	Extraocular motor nuclei [37] Olfactory bulb [38]	IHC, ICC ISH
	Dorsal pontine tegmentum [36]	IHC
EGFR (HER1)	Ovary [41]	Histological ligand binding assay
	Endometrium [42] Placenta [42]	ICC
	Tongue mucosa [43] Transitional epithelium (kidney) [43] Urinary bladder [43] Respiratory epithelium (trachea) [43] Larynx/oral epithelium [43] Peripheral nerve [43]	IHC
HER2 (erbB2)	Mammary gland [44,45] Endometrium [46,47]	IHC
	Uterine wall glandular epithelium [47]	
	Gastric mucosal surface cells and parietal cells (in *Helicobacter heilmanii* colonization) [48]	
RET	Developing gut [49] Developing pancreas [50]	IHC
VEGFR-2 (Flk-1)	Endometrium [51] Placenta [51]	IHC
VEGFR-3	Endothelial cells [52] Mammary gland epithelium [52]	IHC
YES1	Myocardium [53] Adrenal cortex [53] Pancreas [53] Renal tubular epithelium [53] Liver (hepatocytes) [53] Cerebellum [53]	IHC
PDGFR-β	Endocrine pancreas [54] Blood vessel endothelium of normal lymph nodes [54]	IHC
PDGFR-α	Muscle cells [55] Blood vessel walls [55] Hair follicle epithelium [55]	IHC
KIT	Cerebellum [56,57] Endothelial cells [57] Mast cells [56] Mammary gland (weak expression) [56] GI tract, interstitial cells of Cajal [56] Ovary (weak expression) [56] Uterus, endometrium (weak expression) [56]	IHC
	Kidney: cortical arrays, loops of Henle, collecting ducts [58], distal tubules [59]	
	Ureteral wall (interstitial cell of Cajal-like cells) [60] Pancreatic interstitial cells of Cajal [61]	

GI = gastrointestinal; IHC = immunohistochemistry; ISH = in situ hybridization; ICC = immunocytochemistry.

**Table 3:** Summary of the most common adverse events of toceranib phosphate, masitinib mesylate, imatinib mesylate and oclacitinib in cats.

	Toceranib [77,125,129,175,176,177]	Masitinib [139,167,181]	Imatinib [17,19,78,79,161,184]	Oclacitinib [169,170,171,191]
**Most common AE category**	GI (anorexia, vomiting, diarrhea)	GI (vomiting)	GI (vomiting)	GI (vomiting, diarrhea)
	Hepatic (ALT increase)	Renal (proteinuria)	Constitutional (lethargy)	
**Frequency**	47% (GI)	50% (GI)	Occasional	Occasional
	Common (hepatic)	30% (renal)		
**Percent VCOG grade 3 and 4 (out of all cats)**	2% (GI)	0 (GI)	0	0
	6% (hepatic)	30% (renal)		
**Most common dosing schedule**	2.5–2.8 mg/kg q48h or three times per week	50 mg/cat q24h or q48h	10 mg/kg q24h	0.5–2 mg/kg q12h

GI = gastrointestinal; AE = adverse event.

## Data Availability

No new data were created or analyzed in this study. Data sharing is not applicable to this article.
